# Induced pluripotent stem cells in multiple system atrophy: recent developments and scientific challenges

**DOI:** 10.1007/s10286-019-00614-y

**Published:** 2019-06-11

**Authors:** Alain Ndayisaba, Marcos Herrera-Vaquero, Gregor K. Wenning, Nadia Stefanova

**Affiliations:** Division of Neurobiology, Department of Neurology, Medical University of Innsbruck, Innrain 66/G2, 6020 Innsbruck, Austria

**Keywords:** α-Synuclein, iPSCs, Stem cells, Multiple system atrophy, Parkinson’s disease

## Abstract

Multiple system atrophy (MSA) is a rare and fatal neurodegenerative disease, with no known genetic cause to date. Oligodendroglial α-synuclein accumulation, neuroinflammation, and early myelin dysfunction are hallmark features of the disease and have been modeled in part in various preclinical models of MSA, yet the pathophysiology of MSA remains elusive. Here, we review the role and scientific challenges of induced pluripotent stem cells in the detection of novel biomarkers and druggable targets in MSA.

## Introduction

Multiple system atrophy (MSA) is a rapidly progressive and fatal neurodegenerative disease, the etiology of which is currently unknown. Clinically, variable combinations of autonomic dysfunction, parkinsonism, and cerebellar or pyramidal tract dysfunction are observed, and according to the predominance of parkinsonian or cerebellar symptoms are classified into subtype MSA-P or MSA-C, respectively [1]. These syndromes, previously described as the distinct neurological entities Shy-Drager syndrome, olivopontocerebellar atrophy (OPCA), and striatonigral degeneration (SND), have been known under the collective term MSA since 1969 [2], but it was only in 1989 that neuropathological analyses confirmed the presence of so-called Papp–Lantos bodies, glial cytoplasmic inclusions (GCI), in MSA cases [3]. These proteinaceous, primarily oligodendroglial inclusions were shown to be α-synuclein (α-syn)-immunoreactive approximately 20 years ago [4, 5], distinguishing MSA from Parkinson’s disease (PD), dementia with Lewy bodies, and pure autonomic failure, which predominantly exhibit neuronal cytoplasmic and dendritic inclusions containing α-syn as the main component as well [6, 7].

From an epidemiological standpoint, MSA represents an orphan disease with an estimated mean incidence of 0.6–0.7 cases per 100,000 person-years [8]. In the Western Hemisphere, 70–80% of MSA patients are diagnosed with MSA-P [9, 10], whereas in Asian populations MSA-C is found in 67–84%, with a mixed phenotype observed more frequently than in western countries [11, 12]. In patients presenting with parkinsonism or cerebellar ataxia, autonomic failure is a criterion for diagnosis of probable MSA [13]. Currently, diagnosis of definite MSA is made upon postmortem detection of widespread α-syn positive GCIs, the histopathological hallmark of the disease [3], which reflects the difficulty in diagnosing MSA with its variable clinical manifestations [1]. Prior to motor symptom onset, 20–75% of patients experience a prodromal phase, which lasts from several months to years and is characterized by autonomic failure affecting cardiovascular, respiratory, urogenital, gastrointestinal, and sudomotor functions [14]. In addition, rapid eye movement (REM) sleep behavior disorder (RBD) is frequently observed in the premotor stage of α-synucleinopathies, with more than half of patients reporting RBD prior to motor onset, and is present in up to 88% of patients diagnosed with probable MSA [15]. Intriguingly, dermal phospho-α-syn deposits have been detected in isolated RBD cases, whereas no deposits were found in healthy controls [16]. This is supported by a short report by Gaig et al. of one pathologically confirmed MSA case with a long-standing history of stridor, RBD, and autonomic symptoms without parkinsonism or cerebellar signs [17]. Mean age at motor symptom onset is 56.2 ± 8.4 years, with no difference in sex distribution, and median survival is 6–10 (9.8) years [18–20].

The etiology of MSA is still elusive. A complex interaction incorporating genetic predisposition and environmental factors is suggested to drive disease initiation and progression, as familial aggregation following an autosomal dominant or recessive inheritance pattern has been reported in several European and Japanese families [21, 22]. However, MSA is generally considered a sporadic disease with no confirmed risk factors to date [1]. Loss of function mutation in the coenzyme Q2 (COQ2) gene encoding the COQ10-synthesizing enzyme in Japanese familial and sporadic cases and discordant loss of copy numbers of (src homology 2 domain containing)-transforming protein 2 (SHC2) in monozygotic twins and Japanese patients with sporadic MSA have been reported predominantly for MSA-C; however, this was not confirmed in other populations [23–25]. No mutation of the gene coding for α-syn, SNCA, has been found in sporadic MSA; intriguingly, however, oligodendroglial inclusions are detected in cases of familial PD harboring the SNCA mutations [26]. Moreover, clinical features similar to MSA have been observed in some cases [27], indicating a link between oligodendroglial inclusion pathology and MSA phenotype.

## MSA: human postmortem findings

At postmortem examination, neurodegeneration of anatomical areas corresponding to clinical symptoms is observed and therefore varies; however, in cases of predominant parkinsonism, striatonigral degeneration, manifesting macroscopically as atrophy and dark discoloration of the putamen, is found [28, 29]. The cerebellar subtype, on the other hand, presents pathologically with olivopontocerebellar atrophy including the cerebellum, middle cerebellar peduncle, and pontine base [28, 29].

On a cellular basis, MSA is characterized by widespread α-syn immunoreactive inclusion pathology found primarily in oligodendrocytes and to a lesser extent in neurons and other glia. In addition, myelin dysfunction, neuronal loss, and axonal degeneration and microglial activation are present in the brain [28].

The mechanism underlying GCI formation is still uncertain, as mature oligodendrocytes express only low basal levels of α-syn. Hypotheses revolve around (1) increased oligodendroglial α-syn expression in the disease [30], although several studies have failed to show aberrant expression of SNCA mRNA in oligodendrocytes [31–33]; or (2) cell-to-cell transmission of neuronal α-syn to dysfunctional oligodendrocytes not capable of degrading α-syn that has been taken up [34, 35]. In addition, α-syn in MSA also forms glial nuclear inclusions, neuronal cytoplasmic, and nuclear and dendritic inclusions, as well as astroglial cytoplasmic inclusions [36]. In the early stages of neurodegeneration, oligodendroglial dysfunction is observed, which precedes α-syn pathology. Prominent findings at this stage are myelin degeneration reflected by myelin basic protein (MBP) degradation and aberrant composition of myelin lipids, relocation of microtubule polymerization-promoting protein p25α/TPPP to the swollen oligodendroglial soma, and consequently reduced neurotrophic support [28, 37, 38]. GCI density is positively correlated with neuronal loss, and an increase is observed with disease duration [39]. The most severely affected areas include the putamen, caudate nuclei, substantia nigra, pontine and medullary tegmental nuclei, and inferior olive and cerebellar white matter, as well as motor cortex and globus pallidus, and to a lesser extent cingulate cortex, hypothalamus, nucleus basalis of Meynert, thalamus, subthalamus, and pontine tegmentum [40]. More recently, stereological studies reported the occurrence of neocortical atrophy affecting frontal and temporal lobes following degeneration of the basal ganglia [41–43]. Cognitive decline and impaired executive function have been reported in MSA, and it is suggested that focal fronto-striatal degeneration rather that widespread cortical atrophy accounts for the symptoms [44].

Synuclein pathology is suggested to trigger astro- and microglial changes toward an activated and reactive state, which in turn favors neurodegeneration [45]. In MSA brains, more astrocytes and microglia are found in the frontal and parietal cortex, whereas the total number of oligodendrocytes in the neocortex is unaffected [43], reflecting pathological changes consistent with neuroinflammation, one of the drivers of MSA pathogenesis. In contrast to PD, astrogliosis is positively correlated with synuclein pathology in MSA and severity of neurodegeneration [46, 47]. Monoamine oxidase B (MAO-B), a biomarker of astrogliosis, is significantly increased in the putamen (+83%) and correlates positively with α-syn accumulation, whereas a less dramatic increase in MAO-B (+10%) in the substantia nigra correlates with membrane-bound α-syn [48]. MAO-A, on the other hand, is decreased only in the atrophic putamen in the case of MSA (−27%), while in the substantia nigra in PD, no change is observed, thus highlighting aberrant astrocyte behavior in MSA compared to PD. The role of microglia has been studied extensively in neurodegenerative disorders, and yet its full impact on disease pathogenesis is not completely clear, as both pro- and anti-inflammatory properties have been attributed to the ‘macrophages of the brain’, surveying the central nervous system in their resting state. In MSA, microglia become activated in response to an increasing load of misfolded α-syn, which in turn contributes heavily to disease pathogenesis via secretion of pro-inflammatory factors [49]. Microglial activation accompanies GCI pathology in white matter [50], but interestingly, loss of p25α/TPPP immunoreactivity and loss of MBP density correlate strongly with microgliosis as a marker of tract degeneration [51]. Activation of TLR4 and myeloperoxidase has been reported in microglia in MSA [49, 52, 53]. Microglia can be divided into two distinct phenotypes, the pro-inflammatory M1 and the anti-inflammatory M2 phenotype [54]. M1-type microglia is detected more abundantly at the end stage of disease and may represent a consequence of GCIs in MSA [28].

## In vitro and in vivo models of MSA: relevance and limitations

**Fig. 1:**
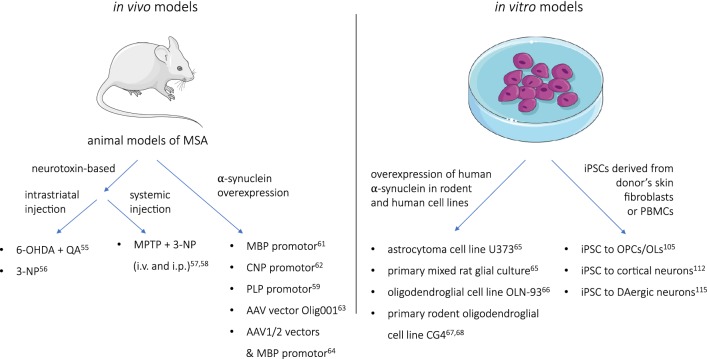
In vivo and in vitro models of MSA. *6-OHDA* 6-hydroxydopamine, *QA* quinolinic acid, *3-NP* 3-nitropropionic acid, *MPTP* 1-methyl-4-phenyl-1,2,3,6-tetrahydropyridine, *i.v.* intravenous, *i.p.* intraperitoneal, *MBP* myelin basic protein, *CNP* 2′,3′-cyclic nucleotide 3′-phosphodiesterase, *PLP* proteolipid protein, *iPSC* induced pluripotent stem cell, *PBMC* peripheral blood mononuclear cell, *OPC* oligodendrocyte progenitor cell, *OL* oligodendrocyte, *DAergic* dopaminergic

Studies on the pathogenic mechanisms of MSA downstream of α-syn aggregation have been carried out in different in vivo and in vitro models, as shown in Fig. 1 [55, 56], in addition to neurotoxin-induced lesions of the striatonigral system [57, 58]. Transgenic oligodendroglial overexpression of α-syn under the proteolipid protein (PLP) promoter results in region-selective neuronal loss associated with early autonomic dysfunction and motor impairment, thereby providing evidence for a causal role of oligodendroglial inclusion formation in the degenerating brain in MSA [59, 60]. The causative role of GCI-like pathology in neurodegeneration has been further supported by studies in additional transgenic models applying alternative oligodendroglial promoters [61–64]. Several in vitro models [65–68] have also been used to study MSA, providing some insight into pathological mechanisms at the molecular level.

These in vivo and in vitro models have been crucial for elucidation of disease mechanisms and continue to represent an invaluable tool for basic research. However, a major limitation in understanding the human disease remains the mechanistic recapitulation of MSA pathology in the available models linked to the lack of knowledge on the initial disease trigger(s).

In recent years, however, the study of patient-specific brain cells derived from easily accessible peripheral tissue via induced pluripotent stem cell (iPSC) technology has flourished, providing a promising template for the study of neurodegenerative diseases and guided drug discovery [69–71].

## Induced pluripotent stem cells as a disease model

Pluripotent stem cells are characterized by their ability to continuously self-renew and to give rise to cells of the three primary germ layers. These so-called embryonic stem cells (ESCs), which occur naturally only in embryos, have been studied since their first derivation from the mouse and human blastocyst [72, 73], with relevance for the modeling of neurodegenerative diseases and development of alternative sources for replacement therapies [74]. However, ethical controversies and limited availability have impeded progress in this field [75]. In 2006, Shinya Yamanaka ushered in a paradigm shift when he showed for the first time the potential for somatic cells to be reprogrammed to a pluripotent state resembling embryonic stem cells, via application of four transcription factors—Oct3/4, Sox2, Klf-4, and c-Myc—which are thus termed induced pluripotent stem cells [76, 77]. Since then, easily accessible peripheral tissue has been used to generate iPSCs by (i) delivery of transcription factors via genome-integrating lenti- or retroviruses; (ii) delivery of transcription factors via non-integrating adenovirus or sendai virus, or (iii) non-viral reprogramming methods including mRNA, miRNA infection/transfection, PiggyBac transposons, minicircle vectors, and episomal plasmids [78]. Subsequent differentiation of patient-derived pluripotent cells reprogrammed into neural cell types has been employed in modeling specific diseases. The directed differentiation of stem cells to specific cell phenotypes is facilitated by the precisely timed addition of molecules influencing cell fate during various stages of neurodevelopment [79].

Midbrain dopaminergic neurons, for example, have been efficiently generated from ESCs [80] and later iPSCs [81–83] to enable modeling of the α-synucleinopathy PD. Although initial differentiation of PD patient-derived iPSCs did not reveal a disease-related phenotype [84], subsequent studies on cell lines harboring PD-causing or PD-associated mutations detected morphological and subcellular changes such as reduced neurite outgrowth, dendrite degeneration and diminished microtubule stability [85–87]. In addition, increased susceptibility to stress of an oxidative or nitrosative nature [87–90], increased levels of α-syn [91, 92] but also elevated α-syn aggregation and Lewy body deposition [90], and mitochondrial dysfunction has been observed [90, 91, 93, 94]. Dopaminergic neurons of individuals with sporadic PD carrying a mutation in the glucocerebrosidase (GBA) gene, show elevated α-syn levels, reduced dopamine storage and uptake, defective autophagic and lysosomal machinery and enhanced vulnerability to endoplasmic reticulum stress [95–97]. Intriguingly, in iPSC-derived neurons of monozygotic twins harboring the GBA N370S mutation and discordant for PD, altered susceptibility toward oxidative stress in the affected twin suggests the presence of disease-contributing factors other than the GBA mutation, which were preserved across the reprogramming and differentiation procedure [96]. Furthermore, researchers reported epigenetic alterations in dopaminergic neurons derived from patients with sporadic PD compared to healthy controls [98].

Efforts have been made to generate specific glial phenotypes. iPSC-derived oligodendrocytes have been generated to support studies on multiple sclerosis [99] and Pelizaeus–Merzbacher disease [100]. Astro- and microglia differentiation protocols have also recently been established [101, 102]. Whether the resultant patient-derived glial cells will reflect features seen in diseased brains remains to be tested.

## iPSC technology to fill the gaps in modeling multiple system atrophy: current developments

In MSA, multiple neuronal and glial phenotypes are affected by the neurodegenerative process linked to α-syn misfolding and accumulation. First efforts have been made to apply the iPSC technology in MSA research (Fig. 1). Recent findings in primary cell cultures suggested a causal role of glia in the pathogenesis of MSA [103]. On the other hand, the origin of α-syn found in MSA oligodendrocytes is still elusive [30, 32, 104]. Djelloul et al. addressed this question by investigating α-syn and SNCA expression in rodent and human models [105]. A primary mixed culture including astrocytes, neurons, oligodendrocytes, and microglia was generated from the postnatal mouse forebrain, and oligodendrocyte progenitors positive for O4 were subsequently isolated. Quantification of α-syn and SNCA transcripts revealed a more than tenfold increase in the oligodendrocyte lineage compared to the whole primary culture. Upon maturation, however, α-syn and SNCA levels decreased substantially as oligodendrocytes started to express maturation markers such as 2′,3′-cyclic-nucleotide 3′-phosphodiesterase (CNPase), galactosylceramide (Gal-C), and MBP. To confirm that this effect was not the result of neuronal–oligodendroglial transfer within the culture, oligodendrocyte precursor cells (OPCs) were generated from mouse ESCs, exhibiting a similar outcome. In a next step, Djelloul et al. applied a modified protocol from Stacpoole et al. [106] to differentiate fibroblast-derived iPSCs from one patient each with MSA-P and MSA-C, one patient suffering from familial PD, and a healthy control into OPCs. After 60 days, oligodendrocyte progenitors—characterized by immunocytochemical confirmation of OPC markers and immature bipolar morphology—revealed α-syn expression in both healthy and diseased lines, with no significant difference between groups. Finally, human oligodendrocyte lineage nuclei were isolated from the pons of three healthy and three MSA postmortem brains to determine the presence of SNCA transcripts, which resulted in the detection of SNCA in one healthy and one diseased sample. This study provides compelling evidence for α-syn expression during oligodendrogliogenesis and subsequent downregulation following maturation in human tissue, complementing previous findings on a physiological occurrence of α-syn in oligodendroglial precursors in animal models [107]. As this downregulation was observed in MSA patient-derived cell lines as well, it is still unclear whether α-syn of oligodendroglial origin does play a role in disease and whether disease-specific features of MSA are partially erased during the whole process of reprogramming and targeted differentiation. Nevertheless, it may be interesting to investigate protein handling of MSA oligodendrocytes and the effects of exogenous addition of α-syn to oligodendrocytes. Kaji et al., for instance, found that the level of endogenous α-syn in primary OPCs from neonatal rats was increased upon the addition of synthetic preformed α-syn fibrils, due to autophagic impairment [108]. Furthermore, the exogenous addition of α-syn led to compromised expression of proteins involved in neuromodulation and myelination, an aspect which, if reproducible in human tissue, may shed great light on MSA pathogenesis.

The contribution of mitochondrial dysfunction to MSA pathogenesis is another aspect currently under discussion. A reduction in respiratory chain complex I activity has been shown in the skeletal muscle of MSA patients [109], whereas platelets and substantia nigra revealed no changes [110]. Mitochondrial dysfunction has also been investigated in MSA mouse models. Studies showed that striatal injection of succinate dehydrogenase inhibitor 3-nitropropionic acid in rats [56] or mitochondrial complex I inhibitor 1-methyl-4-phenylpyridinium ion (MPP+) in mice [111] induced extensive neuronal loss in the substantia nigra and striatum, as well as astrogliosis, accompanied by motor deficits resembling parkinsonism. The strongest evidence to date, however, is from the occurrence of COQ2 mutations in rare Japanese families and sporadic cases presenting predominantly as the cerebellar subtype of MSA [23]. To investigate the effects of functionally impaired variants of COQ2 on mitochondrial function, Nakamoto et al. examined iPSC-derived neurons from a patient with a compound heterozygous COQ2 mutation, an idiopathic MSA patient, and three control lines of diverse descent (Caucasian, African, and Japanese origin) [112]. In addition, an isogenic control was generated by site-specific gene correction of the cell line harboring the COQ2 mutation. In their work, they reprogrammed peripheral blood mononuclear cells into iPSCs, and following a battery of tests to confirm pluripotency and normal karyotype, selected cell clones were differentiated using three different methods to induce neural cells: (i) high-efficiency induction of neurons, (ii) induction of mid-hindbrain neurons [113], and (iii) induction of the three basic lineages of neural cells [114]. Mid- and hindbrain neurons were successfully generated with the latter two methods, representing an area severely affected in MSA-C. Neuronal subpopulations present in the culture included glutamatergic (VGLUT1 and VGLUT2), GABAergic, dopaminergic (tyrosine hydroxylase), and glycinergic (VGAT) neurons.

Mitochondrial integrity, which is expected to be impaired in subjects carrying a mutated COQ2 gene, was assessed by measuring the mean area of the inner mitochondrial membrane, which revealed a significant reduction in the COQ2-mutated MSA patient (MSA_mut) compared with the sporadic MSA patient (MSA_sp) and controls. In addition, COQ10 and vitamin E were quantified and showed reduced levels not only in MSA_mut, but also in the patient cell line carrying the corrected gene (MSA_corr). Changes in mitochondrial respiratory chain activity determined by oxygen consumption rate and extracellular acidification rate showed a significant decrease in MSA_mut and partially in MSA_sp compared to MSA_corr and controls.

Neurodegeneration is observed in the brainstem and striatum of MSA patients postmortem. Analysis of neurite length, however, a sign of neurodegeneration also shown in various iPSC-based models of neurodegeneration, did not reveal any differences between MSA and healthy control lines. Nakamoto and colleagues  then went on to investigate the cellular vulnerability of MSA-derived neurons compared to healthy control neurons using a glucose-free medium and galactose instead, which represents a stress condition. This forces a metabolic switch in cells toward oxidative phosphorylation-dependent ATP production, while glycolysis is inhibited. Staining for cleaved caspase 3 (a marker of apoptosis)-positive neurons in the stress condition was higher in MSA_mut neurons than in controls. MSA_sp showed a tendency for higher levels of apoptosis, and MSA_corr revealed a lower ratio of apoptotic cells compared with MSA_mut, but still significantly higher than in controls. COQ10 supplementation decreased the fraction of apoptotic neurons in MSA_mut, suggesting that low endogenous COQ10 levels are at least partially responsible for the cellular vulnerability observed. In conclusion, this study reveals that the correction of the COQ2 mutation ameliorated mitochondrial function and signs of neurodegeneration but was not able to rescue the cells completely when compared to healthy control lines, indicating that additional factors may come into play here. In addition, the experimental results in cells derived from an idiopathic MSA patient differed significantly from the patient harboring the mutation, therefore additional studies will be needed to identify if this difference is disease-specific or underlies the phenotypic variability between individuals with different genetic background.

Mitochondrial and autophagic dysfunction was also examined in iPSC-derived dopaminergic neurons from MSA patients and healthy controls by Monzio Compagnoni et al. [115]. Cell lines from two MSA-P, two MSA-C, and five healthy controls, among them the healthy monozygotic twin of one of the MSA-C patients (MSA_C1), were generated. In MSA_C2, a homozygous variant in the COQ2 gene (p.A43G) was found, which, according to a previous study using this patient’s cells [116], did not affect respiratory chain activity in muscle or COQ10 levels in muscle and fibroblasts. No other mutations associated with parkinsonism or ataxia were found in any cell line. Upon reprogramming of fibroblasts into iPSCs, dopaminergic neurons were generated according to a protocol by Zhang et al. [117]. To evaluate the maturation and identity of neurons, immunocytochemistry and real time-polymerase chain reaction for markers of dopaminergic neurons was performed, along with electrophysiology to assess post-synaptic activity, and sphingolipid composition was evaluated in three cell lines, confirming neuronal maturation. In order to assess cellular defects present in the MSA lines, western blot analysis of synaptic markers and tau, a neurite protein, was performed and showed decreased levels of synapsin I and tau in MSA patients. Intriguingly, the decrease in tau was not caused by a change in the microtubule-associated protein tau (MAPT) gene levels, and an assessment of α-syn levels did not reveal any differences between MSA and control cell lines. The extent of autophagic impairment was investigated by treating cell cultures with bafilomycin A (a V-ATPase inhibitor which inhibits the fusion of the autophagosome and the lysosome). An increased ratio of LC3-II after treatment to LC3-II basal levels was observed in controls, indicating more efficient autophagic flux, whereas LC3-II basal levels were elevated in MSA lines. Between the twins discordant for the disease, a similar but nonsignificant trend was observed. In addition, the activity of five lysosomal enzymes (GBA1, β-galactosidase, α-mannosidase, β-mannosidase and β-hexosaminidase) was measured, and only α- and β-mannosidase levels were reduced in MSA patients.

Also, mitochondrial dysfunction was investigated by evaluating the activity of respiratory chain complexes I, II, I + III, II + III, and IV by spectrophotometric analysis. The activity of complexes II, III, and II + III was strongly downregulated in MSA patients, but interestingly, the amount of the complexes was not found to be decreased, and was even increased in the case of complexes II and III, conceivably representing a compensatory mechanism. Levels of COQ10 important for mitochondrial activity were normal, yet COQ10-synthesizing enzymes PDSS1 (prenyl (decaprenyl) diphosphate synthase subunit 1), PDSS2, COQ4, and ADCK3 (coenzyme Q8 homolog) were increased. In MSA brain tissue representing the end stage of the disease, however, a decrease in PDSS1 and COQ5 was found [118, 119]. Thus, the authors hypothesize that mitochondrial dysfunction captured in vitro occurs early in disease, and upregulation of COQ10-synthesizing enzymes may represent a compensatory mechanism which fails at later disease stages. In addition, upregulation in MSA fibroblasts from the same group revealed higher levels of COQ5 and COQ7, supporting their findings in dopaminergic neurons [120]. Mitochondrial mass as determined by TOMM20, a mitochondrial structural protein, and mitochondrial DNA content was elevated, leading the authors to hypothesize that those changes in the autophagic system and mitochondria may be linked. They posited that mitochondrial dysfunction could be triggered by insufficient autophagy; thus mitophagy is impaired and senescent, and dysfunctional mitochondria accumulate to promote cellular dysfunction and autophagic dysregulation in a vicious cycle. In a next step, the authors aim to explore α-syn pathological behavior—although α-syn levels were normal in this study—lysosomal dysfunction, and mitophagy.

## Scientific challenges

The first steps indicate the ability of the iPSC technology to reflect disease features and early events in the pathogenesis of MSA. However, first studies point toward significant challenges.

The iPSC-technology is clearly useful for studying the effects of disease-specific mutations in genetic neurodegenerative disorders by correcting the genetic defect with molecular tools [121]. However, sporadic diseases such as MSA lack specific genetic targets, and line-to-line variability due to differences in the genetic background of samples can be expected to hamper scientific interpretation. For years, researchers have tried to pinpoint differences between human ESCs and human iPSCs, but it appears that variability between one iPSC and one ESC line may be smaller than between two iPSC lines [122]. In addition, variability in the epigenetic landscape may result from differences in derivation method and culture history, and differences in cell type of origin may have a substantial impact in subsequent examinations of reprogrammed cells [123]. To overcome these hurdles, sibling lines with similar genetic background may be key for sporadic diseases, as they have been proven to display changes in genetic background despite environmental sources of variability including cell type of origin and derivation method [124]. Quantifiable phenotypic differences between patient and control in vitro in sporadic diseases are expected to be small [121]; therefore, the use of monozygous twin lines discordant for the disease, as in the experiment by Monzio Compagnoni et al., may shed light on disease-specific events. MSA represents a rare disease; therefore, iPSC lines, and especially sibling lines, are very sparse. A combined effort in the form of an international iPSC bio-bank would be instrumental in accelerating MSA research efforts.

As multiple systems and cell types are affected in MSA, co-culture of different cell types may facilitate the study of extracellular disease mechanisms such as α-syn transmission dynamics to answer the question of the origin of GCI-forming α-syn, and may also aid in determining the effects of cellular dysfunction on other cell types at disease onset. The use of more complex models such as 3D brain organoids to study cellular interactions may also be beneficial [125]. Importantly, Madhavan et al. recently demonstrated the generation of cortical oligospheres, an intriguing model for demyelinating diseases and oligodendrogliopathies [126], which may aid in dissecting the complex underlying pathogenesis in MSA. Moreover, both cortical- and midbrain-like organoids have been generated [127, 128] and may prove suitable for the study of MSA in a dish.

## Conclusion

MSA has been studied in various disease models, and despite the elucidation of pathological mechanisms contributing to disease initiation and progression via glial overexpression of α-syn, the underlying pathogenesis of this sporadic disease largely remains an enigma. The emerging iPSC technology offers the unique opportunity to study the disease in the context of patient-specific genetic background. The first studies in MSA patient-specific neural cells revealed mitochondrial, lysosomal, and autophagic dysfunction in iPSC-derived neurons and dysregulation of oligodendroglia consistent with previous findings.

Challenges such as line-to-line variability owing to individual genetic composition might make it difficult to determine whether phenotypic differences between patient and control lines are truly disease-specific, but these may be overcome by increased sample size, technological advancements enabling more sophisticated analysis, and the utilization of sibling lines which exhibit comparable genetic make-up.

In addition, advances in stem cell technology now facilitate the creation of 3D brain structures or organoids and co-culture of multiple cell types to partially reconstruct the complex network of the human brain, which may ultimately aid in identifying druggable targets and their implications in a more sophisticated context, even though it may not be able to replace, but rather complement, functional readouts in in vivo models of neurodegeneration. Nevertheless, iPSC technology represents a promising approach toward two major goals: the detection of novel biomarkers to facilitate earlier diagnosis, and the elucidation of disease-modifying druggable targets.
